# Risk factors for mortality in hospitalized patients with COVID-19 from three hospitals in Peru: a retrospective cohort study

**DOI:** 10.12688/f1000research.51474.1

**Published:** 2021-03-19

**Authors:** Cristian Díaz-Vélez, Diego Urrunaga-Pastor, Anthony Romero-Cerdán, Eric Ricardo Peña-Sánchez, Jorge Luis Fernández Mogollon, Julio Darwin Cossio Chafloque, Gaston Cristobal Marreros Ascoy, Vicente A. Benites-Zapata

**Affiliations:** 1 Universidad Señor de Sipán, Escuela de Medicina, Chiclayo, Peru; 2 Hospital Nacional Almanzor Aguinaga Asenjo, EsSalud, Chiclayo, Peru; 3 Universidad Científica del Sur, Lima, Peru; 4 ADIECS Asociación para el Desarrollo de la Investigación Estudiantil en Ciencias de la Salud, Universidad Nacional Mayor de San Marcos, Lima, Peru; 5 Dirección Ejecutiva de Enfermedades No Transmisibles, Ministerio de Salud, Peru; 6 Hospital Luis Heysen Incháustegui, Chiclayo, Peru; 7 Universidad San Ignacio de Loyola, Unidad de Investigación para la Generación y Síntesis de Evidencias en Salud, Lima, Peru

**Keywords:** SARS-CoV-2, COVID-19, Mortality, Adults, Latin America.

## Abstract

Background: Peru was one of the countries with the highest COVID-19 mortality worldwide during the first stage of the pandemic. It is then relevant to evaluate the risk factors for mortality in patients hospitalized for COVID-19 in three hospitals in Peru in 2020, from March to May, 2020.

Methods: We carried out a retrospective cohort study. The population consisted of patients from three Peruvian hospitals hospitalized for a diagnosis of COVID-19 during the March-May 2020 period. Independent sociodemographic variables, medical history, symptoms, vital functions, laboratory parameters and medical treatment were evaluated. In-hospital mortality was assessed as the outcome. We performed Cox regression models (crude and adjusted) to evaluate risk factors for in-hospital mortality. Hazard ratios (HR) with their respective 95% confidence intervals (95% CI) were calculated.

Results: We analyzed 493 hospitalized adults; 72.8% (n=359) were male and the mean age was 63.3 ± 14.4 years. COVID-19 symptoms appeared on average 7.9 ± 4.0 days before admission to the hospital, and the mean oxygen saturation on admission was 82.6 ± 13.8. While 67.6% (n=333) required intensive care unit admission, only 3.3% (n=16) were admitted to this unit, and 60.2% (n=297) of the sample died. In the adjusted regression analysis, it was found that being 60 years old or older (HR=1.57; 95% CI: 1.14-2.15), having two or more comorbidities (HR=1.53; 95% CI: 1.10-2.14), oxygen saturation between 85-80% (HR=2.52; 95% CI: 1.58-4.02), less than 80% (HR=4.59; 95% CI: 3.01-7.00), and being in the middle (HR=1.65; 95% CI: 1.15-2.39) and higher tertile (HR=2.18; 95% CI: 1.51-3.15) of the neutrophil-to-lymphocyte ratio, increased the risk of mortality.

Conclusions: The risk factors found agree with what has been described in the literature and allow the identification of vulnerable groups in whom monitoring and early identification of symptoms should be prioritized in order to reduce mortality.

## Introduction

COVID-19 is a disease characterized by severe pneumonia, and was first registered in December 2019 in Wuhan, China. ^1^ This disease has generated great impact worldwide, especially in Latin America. ^2^ According to the data reported by the World Health Organization (WHO), the number of COVID-19 cases at the end of December 2020 exceeded 80 million around the world, while fatal cases amount to more than 1.7 million. ^2^ However, in regions with emerging economies or with limited access to health services such as Latin America, this disease has had great social impact. ^3^

It is known that approximately 80% of COVID-19 cases present a mild to moderate course; however, the remaining 20% present a severe to critical course, requiring hospital care and leading to a high risk of death. ^4^ Thus, factors affecting the prognosis of this disease have been related to the severity of the clinical presentation and analytical markers. The analytical factors include a high neutrophil-to-lymphocyte ratio (NLR), platelet-to-lymphocyte ratio and lymphocyte-to-monocyte ratio, ^5^ leukopenia, elevated creatinine and lactate dehydrogenase (LDH) levels and prothrombin time. ^6^ On the other hand, demographic characteristics and medical history have been described to increase the risk of mortality due to COVID-19 and include advanced age, male sex, the presence of comorbidities, such as chronic obstructive pulmonary disease, hypertension, type 2 diabetes mellitus or coronary heart disease. ^6, 7^

Although risk factors for the disease have been described in different populations around the world, there is little scientific evidence in the Latin American population and especially in Peru, which is one of the countries with the highest mortality from COVID-19 worldwide. ^8, 9^ In addition, deficiencies in the Peruvian health system, including a scarcity of intensive care unit (ICU) beds and mechanical ventilators, as well as low compliance with government measures to combat the spread of the pandemic, could increase mortality by COVID-19. ^8, 10^ Likewise, the massive use of therapies without scientific evidence in the hospital setting, as well as the high frequency of self-medication in the Peruvian population can also aggravate the severity and mortality by COVID-19. ^11^

Despite the government having taken measures to improve access to health services in Peru, the implementation of these strategies was slow during the first months of the pandemic. The impact of the flaws of the health system could have caused a higher mortality in the population. Taking this into account, it is relevant to explore the sociodemographic and clinical characteristics of the population hospitalized for COVID-19 in Peru and the incidence of mortality at the first stage of the pandemic. Therefore, the objective of this study was to evaluate and describe the risk factors for mortality from COVID-19 in patients from three hospitals in Peru hospitalized during the period from March to May 2020.

## Methods

### Design and population

We carried out a retrospective cohort study. The study population consisted of adults hospitalized during the period from March 18 to May 13, 2020 for the diagnosis of COVID-19 at the Hospital Almanzor Aguinaga Asenjo and Luis Heysen Incháustegui Hospital, located in Chiclayo and the Hospital Clínica EsSalud Chepén, located in Chepén, two cities in Peru. We included patients older than 18 years of age who were hospitalized with a diagnosis confirmed by serological or molecular tests for COVID-19, as well as suspected by a compatible clinical or radiological pattern plus an epidemiological link, despite having a non-reactive serological test for COVID-19. The exclusion criteria were patients under 18 years of age and pregnant women.

### Description of the study area

The hospitals included correspond to the Lambayeque social security care network in Peru (EsSalud) and are the reference hospitals with the highest complexity for the management of patients with COVID-19. From the first confirmed case of COVID-19 in Lambayeque in March 2020 until November 30, 2020, there was a total of 18,570 confirmed cases of COVID-19, 5,654 hospitalized cases and 2,579 deaths.

### Type of sampling and sample size calculation

The sampling was non-probabilistic and we included all participants hospitalized during the study period who met the inclusion criteria.

### Procedures

The information on the participants included in the study was collected by two researchers from the EsSalud virtual medical records registry. The collection of all records was carried out independently by each of the data entry operators, to ensure adequate data collection and reduce erroneous records. Sociodemographic variables, medical history and laboratory markers were collected from hospitalized patients during the period from March 18 to May 13, 2020. Laboratory markers were collected during the first 24 hours of hospitalization. Mortality follow-up was in-hospital and the date of hospital admission was considered as the start of follow-up. The information was tabulated in a Microsoft Excel 2016 document, and quality control of the data was carried out by a researcher of our team.

### Bias

This study included participants insured by Peruvian social security, who have socioeconomic characteristics that could differ from the national population; however, this study is one of the first reports carried out in Peru, ^12, 53^ one of the countries with the highest mortality rates during the first wave of the pandemic. In addition, certain laboratory markers had a significant percentage of missing values, however, we included in the multivariate analysis the most relevant markers for the association of interest.

### Variables

Outcome variable: In-hospital mortality

Mortality in hospitalized patients with a confirmed or probable diagnosis of COVID-19 was evaluated as an outcome variable. This was collected according to outcome recorded in the clinical history at the end of the follow-up (June 2, 2020).

Exposure variables

Sociodemographic variables

The demographic characteristics recorded were: age (<50, 50-59, ≥60 years), sex (female, male), comorbidities (obesity, type 2 diabetes mellitus, hypertension, asthma, cancer, chronic kidney disease). Likewise, we generated a variable that grouped these comorbidities into different categories (0, 1, 2 or more).

Symptoms and epidemiological link

The time of disease of the patients (in days), symptoms (respiratory distress, cough, fever, sore throat, diarrhea, headache, nasal congestion, anosmia, ageusia) were included. In addition, contact with a confirmed case of COVID-19 (yes, no) was considered.

Baseline vital functions

Baseline vital function values were collected at admission, including temperature, respiratory rate, heart rate, and oxygen saturation.

Baseline auxiliary exams

The following laboratory values were considered: hemoglobin (g/dL), leukocytes (leukocytosis was defined as a value greater than or equal to 10,000 cells/mm ^3^), neutrophils (cells/mm ^3^), lymphocytes (lymphopenia was defined as a value less than 0.8 cells/mm ^3^), the NLR (categorized into tertiles), platelets (thrombocytopenia was defined as a value less than 150,000 cells/mm ^3^), creatinine (mg/dL), urea (mg/dL), aspartate transaminase (AST) (U/L), alanine aminotransferase (ALT) (U/L), and LDH (U/L).

Treatment received

The treatment administered to hospitalized patients was included, considering antibiotic therapy (azithromycin, cephalosporins, carbapenems, among others), corticosteroid therapy (methylprednisolone, dexamethasone, hydrocortisone, prednisone), antiparasitic drugs (hydroxychloroquine, ivermectin), anticoagulants (enoxaparin) and antivirals (lopinavir)/ritonavir).

### Statistical analysis

The descriptive results of the categorical variables were presented using absolute and relative frequencies, while quantitative variables are shown using central tendency and dispersion measures. The comparison of proportions between the categorical covariates and the outcome was performed using the Chi-square test, while the Student's t test or Mann Whitney U test was used to evaluate differences with numerical covariates.

The Kaplan-Meier method was used to describe the survival function, and the log-rank test was used for the crude comparison of survival functions. A Cox regression analysis (crude and adjusted) was performed to evaluate independent risk factors for mortality in the study sample. The adjusted model included variables the association of which has been described in the literature. ^6, 12^ Crude and adjusted hazard ratios (HR) were calculated with their respective 95% confidence intervals (95% CI). Compliance with the proportionality of hazards assumption of the Cox model was verified and collinearity relationships were evaluated in the adjusted model. All analyses were conducted with the statistical package STATA v14.0.

### Ethical aspects

This study was carried out following the guidelines of the Declaration of Helsinki of 1964 and its subsequent amendments. The virtual medical records were reviewed without affecting the social, psychological and physical integrity of the study participants. We did not request the signing of an informed consent because the data was anonymized and we did not violate the integrity of the participant. In addition, this study was evaluated and approved by the Research Ethics Committee for COVID-19 of EsSalud, Peru (N°42-IETSI-ESSALUD-2020).

## Results

### Descriptive and bivariate analyses according to mortality in the study sample.

A total of 493 hospitalized adults were analyzed, 72.8% (n=359) of whom were male with a mean age of 63.3 ± 14.4 years. Likewise, 62.5% (n=308) were 60 years of age or older, 25% (n=123) had hypertension, and 16.5% (n=81) were obese. The median length of hospital stay was five days (IQR: 3-9), and symptoms appeared on average 7.9 ± 4.0 days before admission to the hospital, the most common being respiratory distress, followed by cough and fever, while only 3.7% (n=18) reported having had contact with a confirmed case of COVID-19. Likewise, the mean oxygen saturation upon hospital admission was 82.6 ± 13.8; 83.8% (n=413) of the cases were confirmed, but only 3.4% (n=14) of the confirmed cases were diagnosed by a real-time polymerase chain reaction (RT-PCR) test. 61.1% (n=258) of the participants had leukocytosis, and 39.6% (n=167) had lymphopenia. While 67.6% (n=333) required ICU admission, only 3.3% (n=16) were actually admitted to this unit, and 60.2% (n=297) of the sample died. In addition, there were 1.99 deaths per 100 person-days at risk. Table 1 shows the bivariate analysis of the study variables and mortality.

**Table 1.** Descriptive and bivariate analysis according to in-hospital death in the study sample.

Variables	n	%	mean ± SD ^1^	In-hospital death		
				Survivor	Non-survivor	P value
				n=196 (39.8%)	n=297 (60.2%)	
**Demographic characteristics**						
Age			63.3 ± 14.4	56.4 ± 13.4	67.9 ± 13.1	**<0.001**
<50 years	85	17.2		58 (68.2)	27 (31.8)	**<0.001**
50-59 years	100	20.3		54 (54.0)	46 (46.0)	
≥60 years	308	62.5		84 (27.3)	224 (72.7)	
Sex						0.638
Female	134	27.2		51 (38.1)	83 (61.9)	
Male	359	72.8		145 (40.4)	214 (59.6)	
Comorbidities						**<0.001**
0	267	54.2		115 (43.1)	152 (56.9)	
1	143	29.0		63 (44.1)	80 (55.9)	
2 or more	83	16.8		18 (21.7)	65 (78.3)	
Obesity	81	16.5		28 (34.6)	53 (65.4)	0.297
Type 2 diabetes mellitus	91	18.5		31 (34.1)	60 (65.9)	0.219
Hypertension	123	25.0		39 (31.7)	84 (68.3)	**0.035**
Asthma	14	2.8		5 (35.7)	9 (64.3)	0.754
Cancer	11	2.2		3 (27.3)	8 (72.7)	0.539
Chronic kidney disease	10	2.0		2 (20.0)	8 (80.0)	0.328
**Symptoms and epidemiological link**						
Time of disease (n=405)			7 (5-10)	7 (6-10)	7 (5-10)	0.295
Symptoms (n=450)						
Breathing distress	407	90.4		165 (40.5)	242 (59.5)	0.449
Cough	357	79.3		148 (41.5)	209 (58.5)	0.770
Fever	246	54.7		104 (42.3)	142 (57.7)	0.581
Sore throat	48	10.7		22 (45.8)	26 (54.2)	0.482
Diarrhea	40	8.9		21 (52.5)	19 (47.5)	0.125
Headache	30	6.7		15 (50.0)	15 (50.0)	0.306
Nasal congestion	9	2.0		2 (22.2)	7 (77.8)	0.319
Anosmia	3	0.7		2 (66.7)	1 (33.3)	0.571
Ageusia	2	0.4		1 (50.0)	1 (50.0)	1.000
Contact with a confirmed case of COVID-19						
Yes	18	3.7		7 (38.9)	11 (61.1)	0.939
**Baseline vital functions**						
Temperature (°C) (n=284)			36.8 (36.6-37.2)	36.8 (36.6-37.4)	36.8 (36.5-37.0)	0.299
Fever (≥38 °C)	30	10.6		16 (53.3)	14 (46.7)	0.476
Respiratory rate (n=402)			26 (22-31)	24 (22-28)	28 (24-32)	**<0.001**
Tachypnea, ≥22	334	83.1		132 (39.5)	202 (60.5)	**0.023**
Tachypnea, ≥30	136	33.8		34 (25.0)	102 (75.0)	**<0.001**
Heart rate (n=467)			91 (82-108)	88 (80-100)	95 (84-110)	**<0.001**
Tachycardia, ≥100	176	37.7		49 (27.8)	127 (72.2)	**<0.001**
Tachycardia, ≥120	46	9.9		10 (21.7)	36 (78.3)	**0.008**
Oxygen saturation, % (n=470)			88 (78-92)	90 (87-93)	82.5 (70-89.5)	**<0.001**
<96%	432	91.9		165 (38.2)	267 (61.8)	**0.001**
<94%	403	85.7		143 (35.5)	260 (64.5)	**<0.001**
<92%	347	73.8		111 (32.0)	236 (68.0)	**<0.001**
<90%	278	59.2		68 (24.5)	210 (75.5)	**<0.001**
<85%	181	38.5		23 (12.7)	158 (87.3)	**<0.001**
<80%	125	26.6		11 (8.8)	114 (91.2)	**<0.001**
**Case definition and diagnosis**						
Positive diagnosis (n=413)						0.547
Rapid serological test positive to IgG	19	4.6		7 (36.8)	12 (63.2)	
Rapid serological test positive to IgM/IgG	343	83.0		139 (40.5)	204 (59.5)	
Rapid serological test positive to IgM	37	9.0		15 (40.5)	22 (59.5)	
RT-PCR	14	3.4		3 (21.4)	11 (78.6)	
Case definition						0.961
Suspicious	80	16.2		32 (40.0)	48 (60.0)	
Confirmed	413	83.8		164 (39.7)	249 (60.3)	
**Baseline auxiliary exams**						
Hemoglobin, g/dL (n=333)			13.4 (12.4-14.3)	13.8 (12.6-14.6)	13.3 (12.3-14.1)	**0.0198**
Leukocytes, cells/mm^3 (n=422)			11.6 (8.3-15.6)	9.1 (6.9-12.6)	13.0 (9.9-17.5)	**<0.001**
Leukocytosis (≥10,000 cells/mm^3)	258	61.1		77 (29.8)	181 (70.2)	**<0.001**
Neutrophils, cells/mm^3 (n=422)			9.6 (6.8-14.0)	7.5 (5.3-11.1)	111	**<0.001**
Lymphocytes, cells/mm^3 (n=422)			0.92 (0.6-1.3)	1.0 (0.7-1.4)	0.8 (0.6-1.2)	**<0.001**
Lymphopenia (<0.8 cells/mm^3)	167	39.6		51 (30.5)	116 (69.5)	**<0.001**
NLR (n=422)			11 (6.5-18.2)	7.7 (4.6-12.7)	12.9 (8.7-23.3)	**<0.001**
Low tertile	141	33.4	4.9 (3.5-6.5)	92 (65.3)	49 (34.7)	**<0.001**
Intermediate tertile	141	33.4	11 (9.4-12.6)	56 (39.7)	85 (60.3)	
High tertile	140	33.2	23.5 (18.2-31.7)	32 (22.9)	108 (77.1)	
Platelets, cells/mm^3 (n=422)			286.1 ± 124.2	295.1 ± 125.2	279.3 ± 123.2	0.196
Thrombocytopenia (<150,000 cells/mm^3)	47	11.1		17 (36.2)	30 (63.8)	0.340
Creatinine, mg/dL (n=350)			0.74 (0.59-0.92)	0.71 (0.57-0.84)	0.75 (0.60-0.97)	**0.025**
Urea, mg/dL (n=189)			39.2 (28.0-53.0)	36.5 (27.2-45.8)	46.6 (30.3-71.0)	**<0.001**
AST, U/L (n=195)			41.0 (30.0-58.9)	40 (27-57)	42.2 (32.0-58.9)	0.262
ALT, U/L (n=203)			42 (26-73)	50.3 (24.4-81.0)	39.9 (26.7-61.0)	0.259
LDH, U/L (n=166)			403 (302-530)	302 (225-367)	484 (409-697)	**<0.001**
LDH ≥245 U/L	146	88.0		50 (34.3)	96 (65.8)	**<0.001**
LDH ≥450 U/L	69	41.6		4 (5.8)	65 (94.2)	**<0.001**
**Time**						
Hospital stay length, days			5 (3-9)	8 (5-11)	3 (2-6)	**<0.001**
**Outcomes**						
Admitted to ICU	16	3.3		3 (18.8)	13 (81.3)	0.081
High Flow oxygen requirement (FiO2 ≥0.36)	350	71.0		60 (17.1)	290 (82.7)	**<0.001**
ICU requirement (FiO2 ≥0.80)	333	67.5		47 (14.1)	286 (85.9)	**<0.001**

Data expressed as mean ± standard deviation, median (interquartile range) or number (percentage). ^1^ SD: Standard deviation.

AST: aspartate transaminase; ALT: alanine aminotransferase; LDH: lactate dehydrogenase; ICU: intensive care unit; RT-PCT: real-time polymerase chain reaction; NLR: neutrophil-to-lymphocyte ratio.

HR: Hazard ratio; cHR: crude hazard ratio; aHR: adjusted hazard ratio; 95% CI: 95% confidence interval; NLR: neutrophil-to-lymphocyte ratio.

### Treatment received by the study participants

Antibiotic therapy was administered to 98.8% (n=487) of the participants, including a combination of azithromycin + cephalosporins in 78.2% (n=381). Likewise, 65.7% (n=324) of the participants received corticosteroid treatment, with methylprednisolone being the preferred treatment in 64.8% (n=210), followed by dexamethasone with 27.2% (n=88). Additionally, 76.7% (n=378) of the sample received hydroxychloroquine, and 26.0% (n=128) received ivermectin, while 79.3% (n=391) were prescribed enoxaparin. Likewise, in the bivariate analysis, statistically significant differences were found between the types of corticosteroids received, having received enoxaparin, and mortality ( Table 2).

**Table 2.** Descriptive and bivariate analysis of the treatment received according to in-hospital death in the study sample.

Variables	n	%	In-hospital death		
			Survivor	Non-survivor	P value
			n=196 (39.8%)	n=297 (60.2%)	
**Received antibiotic therapy**					0.686
No	6	1.2	3 (50.0)	3 (50.0)	
Yes	487	98.8	193 (39.6)	294 (60.4)	
**Types of antibiotic therapy**					0.919
Azithromycin+ Cephalosporins	381	78.2	151 (39.6)	230 (60.4)	
Azithromycin	45	9.3	20 (44.4)	25 (55.6)	
Cephalosporins	26	5.3	10 (38.5)	16 (61.5)	
Azithromycin + Carbapenems	23	4.7	7 (30.4)	16 (69.6)	
Carbapenems	6	1.2	3 (50.0)	3 (50.0)	
Azithromycin + others	4	0.8	1 (25.0)	3 (75.0)	
Others	2	0.4	1 (50.0)	1 (50.0)	
**Received corticosteroids**					0.971
No	169	34.3	67 (39.6)	102 (60.4)	
Yes	324	65.7	129 (39.8)	195 (60.2)	
**Type of corticosteroids**					**<0.001**
Methylprednisolone	210	64.8	72 (34.3)	138 (65.7)	
Dexamethasone	88	27.2	48 (54.6)	40 (45.4)	
Hydrocortisone	21	6.5	4 (19.1)	17 (80.9)	
Prednisone	5	1.5	5 (100.0)	0 (0.0)	
**Received hydroxychloroquine**					0.554
No	115	23.3	43 (37.4)	72 (62.6)	
Yes	378	76.7	153 (40.5)	225 (59.5)	
**Received ivermectin**					0.283
No	365	74.0	140 (38.4)	225 (61.6)	
Yes	128	26.0	56 (43.8)	72 (56.2)	
**Received enoxaparin**					**<0.001**
No	102	20.7	55 (53.9)	47 (46.1)	
Yes	391	79.3	141 (36.1)	250 (63.9)	
**Received Lopinavir/Ritonavir**					0.283
No	367	74.4	151 (41.1)	216 (58.9)	
Yes	126	25.6	45 (35.7)	81 (64.3)	

### Survival estimated using Kaplan-Meier curves

**Figure 1.**
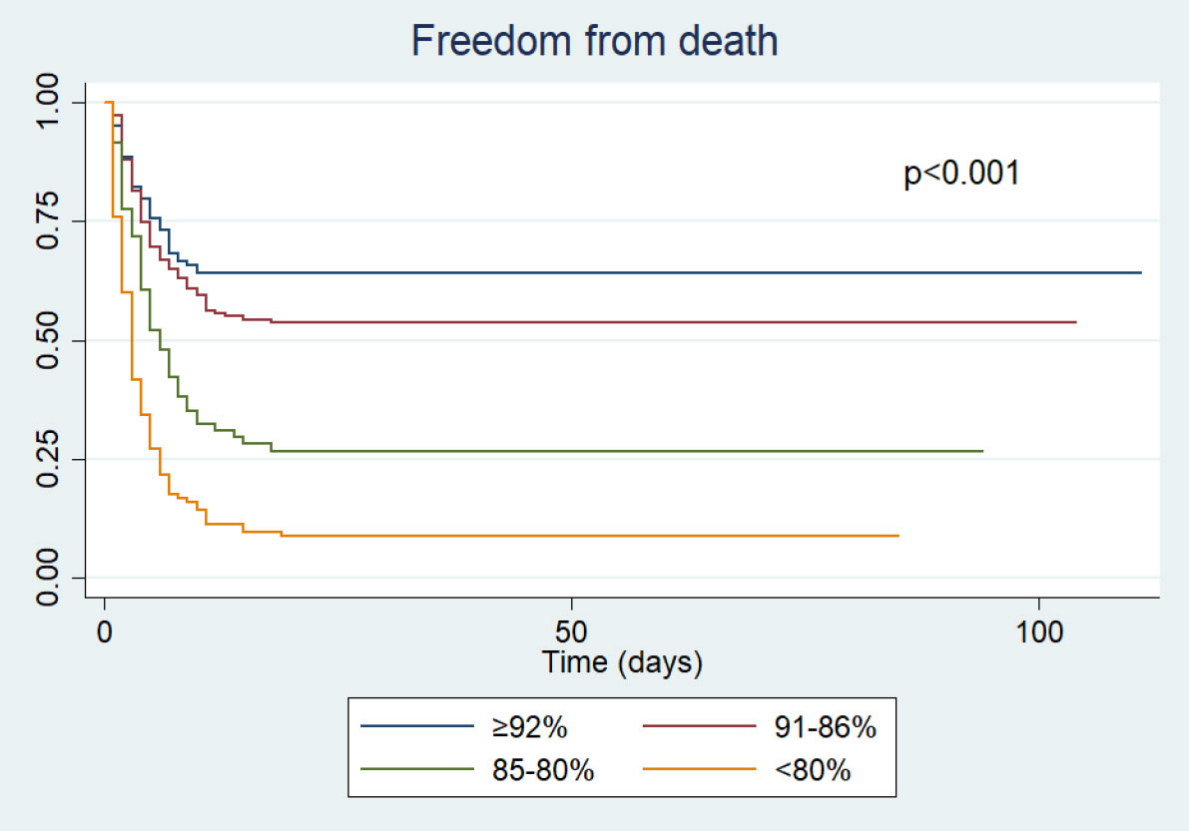
Survival analysis by oxygen saturation level at hospital admission.

**Figure 2.**
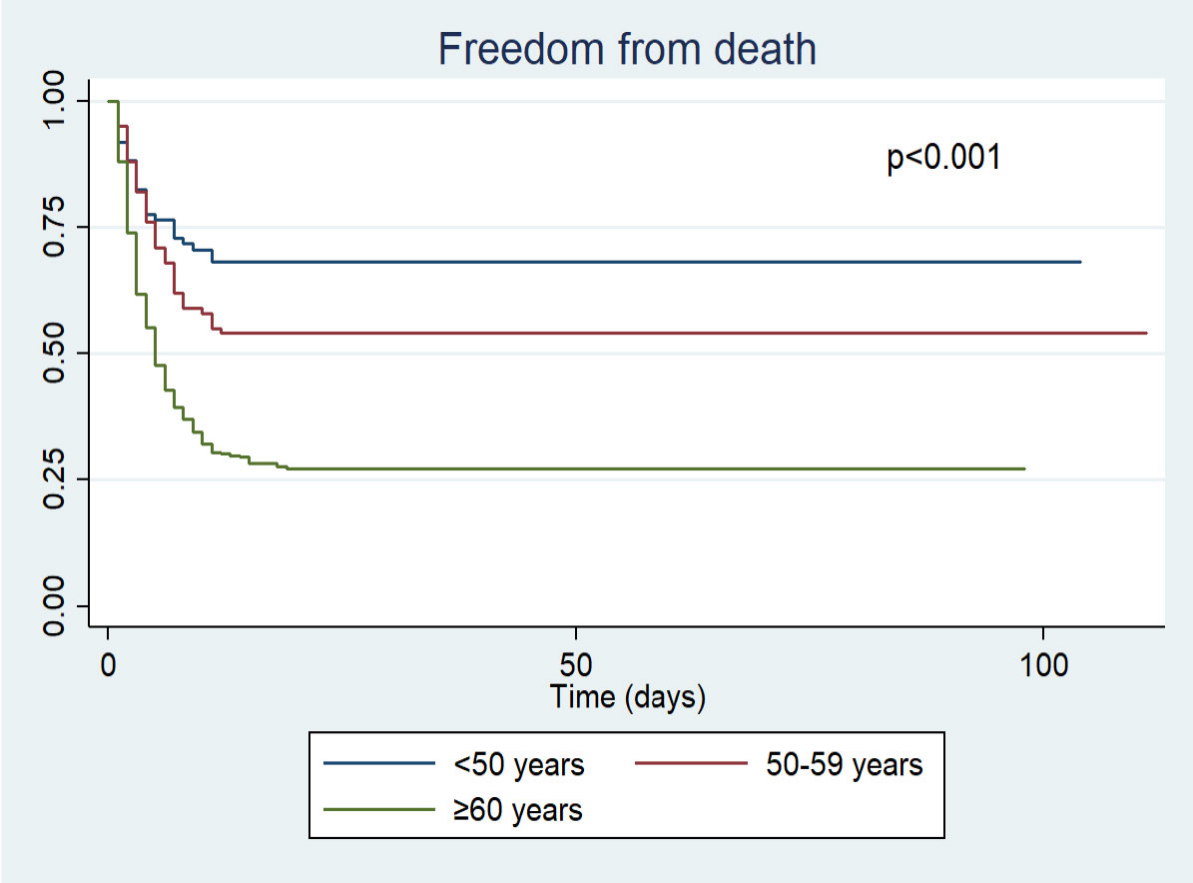
Survival analysis by age groups of the participants.

**Figure 3.**
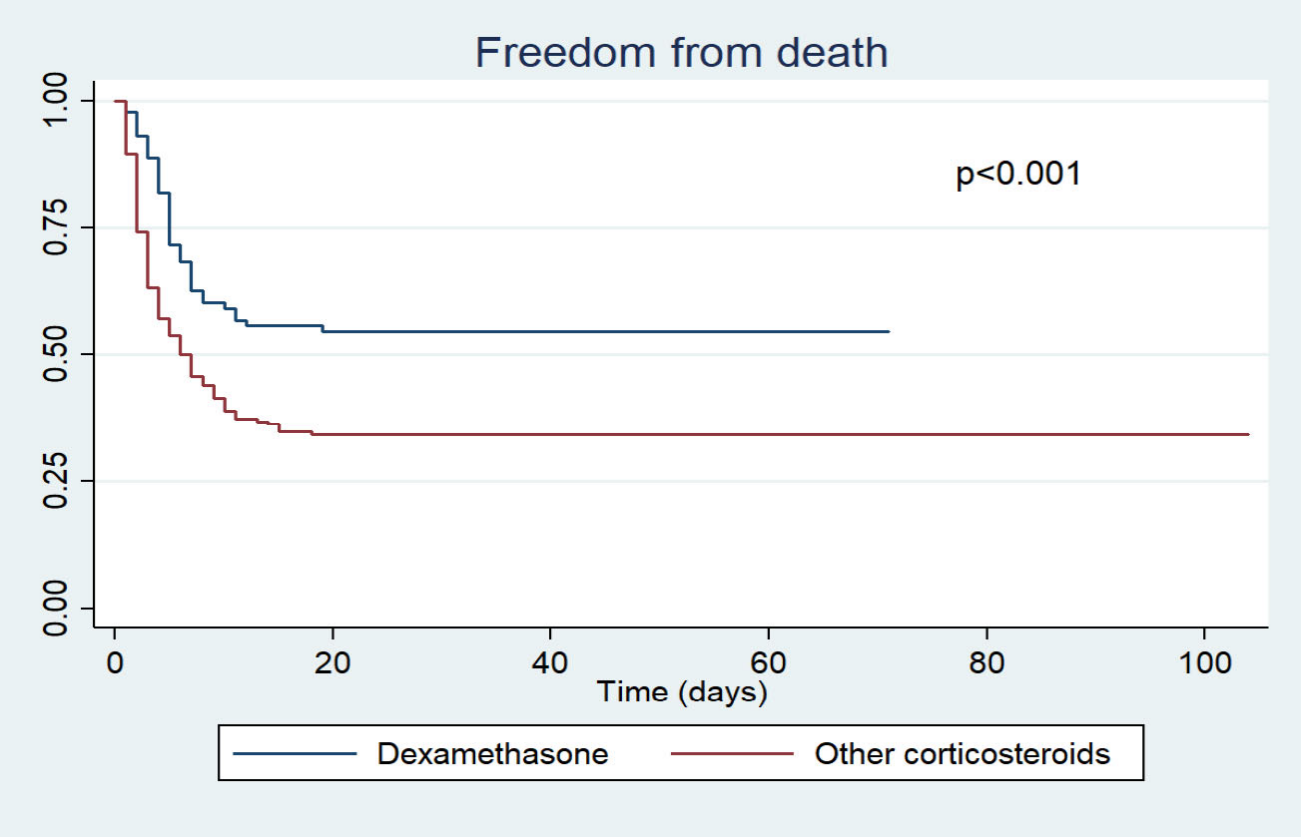
Survival analysis by group of corticosteroids received.

A better survival curve was found in participants admitted to hospital with a higher oxygen saturation (≥92% vs. 91-86% vs. 85-80% vs. <80%), which was statistically significant (p<0.001) ( Figure 1). Likewise, the survival curve was better in younger patients (<50 years vs. 50-59 vs. ≥60) and in those who received dexamethasone (dexamethasone vs. others) during hospitalization ( Figures 2 and 3). These differences were statistically significant (p<0.001).

### Risk factors for mortality in hospitalized COVID-19 patients in the study sample

In the crude Cox regression analysis, it was found that age greater than or equal to 60 years (crude hazard ratio [cHR]=2.52; 95% CI: 1.93-3.28), having two or more comorbidities (cHR=1.59; 95% CI: 1.19-2.12), oxygen saturation between 85-80% (cHR=2.70; 95% CI: 1.81-4.04) or less than 80% (cHR=5.25; 95% CI: 3.69-7.46), as well as the intermediate (cHR=2.16; 95% CI: 1.52-3.07) and high NLR tertile (cHR=3.40; 95% CI: 2.42-4.78) were associated with a higher risk of mortality in hospitalized COVID-19 patients in Peru. In the adjusted regression analysis, age greater than or equal to 60 years (adjusted hazard ratio [aHR]=1.57; 95% CI: 1.14-2.15), having two or more comorbidities (aHR=1.53; 95% CI: 1.10-2.14), oxygen saturation between 85-80% (aHR=2.52; 95% CI: 1.58-4.02), less than 80% (aHR=4.59; 95% CI: 3.01-7.00), as well as being in the intermediate (aHR=1.65; 95% CI: 1.15-2.39) and higher tertile NLR (aHR=2.18; 95% CI: 1.51-3.15) remained associated with a higher risk of mortality ( Table 3).

**Table 3.** Cox regression analysis to evaluate risk factors of mortality in the study sample.

Variables	Crude model		Adjusted model	
	cHR (95% CI)	P value	aHR (95% CI)	P value
Age				
≥60 years	**2.52 (1.93-3.28)**	**<0.001**	**1.57 (1.14-2.15)**	**0.006**
Sex				
Female	Reference		Reference	
Male	0.94 (0.73-1.21)	0.645	1.05 (0.77-1.42)	0.756
Comorbidities				
0	Reference		Reference	
1	0.92 (0.70-1.21)	0.559	0.92 (0.67-1.26)	0.602
2 or more	**1.59 (1.19-2.12)**	**0.002**	**1.53 (1.10-2.14)**	**0.011**
Oxygen saturation				
≥92%	Reference		Reference	
91-86%	1.35 (0.93-1.97)	0.116	1.18 (0.75-1.84)	0.474
85-80%	**2.70 (1.81-4.04)**	**<0.001**	**2.52 (1.58-4.02)**	**<0.001**
<80%	**5.25 (3.69-7.46)**	**<0.001**	**4.59 (3.01-7.00)**	**<0.001**
NLR tertiles				
Low tertile	Reference		Reference	
Intermediate tertile	**2.16 (1.52-3.07)**	**<0.001**	**1.65 (1.15-2.39)**	**0.007**
High tertile	**3.40 (2.42-4.78)**	**<0.001**	**2.18 (1.51-3.15)**	**<0.001**

## Discussion

### Main results

This study included 493 patients hospitalized in three hospitals in Peru. We found that approximately six out of 10 patients died during follow-up, and while about seven out of 10 required admission to the ICU, only 3.3% were actually admitted to this unit. In addition, it was found that being an older adult, having an oxygen saturation level of 85% or less at admission, and having a high NLR value were associated with a higher risk of mortality. In addition, approximately eight out of 10 hospitalized patients received hydroxychloroquine and nine out of 10 were prescribed azithromycin.

It was found that about six in 10 hospitalized patients died, which is a high frequency compared to the 28.3%, 21.7% and 14.6% reported in patients hospitalized for COVID-19 from Wuhan, ^6^ New York ^13^ and Madrid, ^14^ respectively. Likewise, the mortality found in this study was higher than the 39.6% and 38% described in studies carried out in Brazil ^15^ and Mexico, ^16^ respectively. This could be due to patients having received hospital care on average 7.9 days after the onset of symptoms, with a subsequently higher risk of severe disease and mortality. Likewise, it was of note that 73.8% of the patients arrived at the hospital with an oxygen saturation lower than 92%, hypoxia being a risk factor for mortality. ^17^ On the other hand, despite 67.5% of the patients requiring admission to the ICU, only 3.3% were actually admitted, which could explain the high mortality and the shorter hospital stay in the group that died. In the present study, approximately 25% of the deaths occurred within the first 24 hours of hospitalization, suggesting that the population arrived late to medical care and adequate early monitoring of symptoms, which could avoid complications, was not carried out. ^18^

In this study, older adults were found to have a higher risk of mortality. This situation is consistent with what has been described in previous studies, ^6, 19^ and could be explained by greater dysregulation of immune function and immunosenescence in older adults, as well as a higher prevalence of comorbidities. ^20^ Certain comorbidities, such as hypertension, diabetes mellitus and chronic kidney disease, are treated with angiotensin converting enzyme inhibitors and angiotensin II receptor blockers, increasing the risk of severe disease and mortality. ^19^ However, the role of the immune system in the pathophysiology of COVID-19 in this age group is still under study.

Furthermore, elevated NLR values were found to be associated with an increased risk of mortality. Inflammation plays a relevant role in the pathophysiology of COVID-19 and allows establishing the prognosis of patients. Within the response of the innate immune system to a respiratory infection, there is a proliferation of neutrophils at the alveolar level, which could generate collateral damage and cytotoxicity. Furthermore, the release of anti-inflammatory cytokines could lead to lymphocyte apoptosis, producing lymphocytopenia. ^21, 22^ In this way, an elevated NLR has been described as an indicator of severe inflammation progression, which could lead to complications such as sepsis, multi-organ failure, and acute respiratory distress syndrome. ^23^ In previous studies, it has been described as a prognostic marker for COVID-19, and its usefulness is highlighted due to its low cost, easy implementation and practicality. ^24^

Hypoxemia was found to be a risk factor for mortality in the study sample, which is similar to results in previous studies. ^25, 26^ Approximately four out of 10 hospitalized patients arrived at the hospital with oxygen saturation lower than 86%, indicating that these patients arrived late at the hospital and correlates with the mean time of disease onset that exceeded seven days. Thus, the high proportion of patients with hypoxemia could be associated with the high mortality in the sample. Likewise, patients who arrive at the hospital with a higher degree of hypoxia require more intensive care, oxygen support, access to the ICU and mechanical ventilation, ^17^ which in Peru is limited ^27^ and could explain the high incidence of mortality. It should be noted that hypoxia has been associated with inflammation, which with the proliferation and elevation of cytokine levels, increases the already established lung damage and worsens the prognosis. ^28^

We found an association between having two or more comorbidities and a higher risk of mortality. The main comorbidities in the population were hypertension, diabetes mellitus and obesity, which have been described as predictors of severity and worse prognosis in previous studies. ^29– 31^ The pro-inflammatory role of obesity has been mentioned, which induces diabetes mellitus and oxidative stress, affecting cardiovascular function. ^32^ Likewise, a greater abdominal circumference increases breathing difficulty, which can restrict ventilation by decreasing the excursion of the diaphragm. ^33^ It should be noted that diabetes mellitus and obesity alter immune response to viral infections. ^33^

Nearly all of the participants received antibiotic therapy, the main drug being azithromycin. However, less than 7% of patients hospitalized for COVID-19 were reported as having bacterial coinfection. ^34^ Likewise, the use of azithromycin in conjunction with hydroxychloroquine gained relevance based on initial favorable reports in March 2020, ^35^ both drugs being approved for use in Peru as of April 2020. ^36^ However, later, the use of these drugs was rejected internationally, due to their null positive effect ^37, 38^ and the increased risk of mortality. ^39^ On the other hand, another drug frequently used was ivermectin, which began to gain relevance within the scheme of the Ministry of Health of Peru (MINSA) due to an *in vitro* study published during the study period. ^40^ However, this drug was consolidated in Peru over the following months after promoting its use through self-medication ^41^ and even subdermal application. ^42^ The use of these medical therapies based on studies with biases or design flaws ^35, 43^ could have caused people to have false security and to have not gone to the hospital on the appearance of symptoms, thereby leading to disease progression and an increased risk of death or the development of severe illness. On the other hand, corticosteroids were administered in two out of three people, with a predominance of methylprednisolone, while dexamethasone, which was reported to reduce mortality in hospitalized COVID-19 patients in June, ^44^ was not the most widely used.

The health system in Peru is fragmented and is currently overwhelmed. ^45^ Although progress has been made in universal insurance for the population, ^46^ the designated health budget is 2.3% of the annual gross domestic product. ^47^ Moreover, despite the low budget assigned, it is not fully executed annually, ^47^ which could further explain the shortcomings of the health system. In Peru, there were approximately 0.2 ICU beds per 100,000 inhabitants before the pandemic, ^27^ which, in addition to the deficit of oxygen and hospital beds, could explain the high mortality that led Peru to occupy the first place in mortality per 100,000 inhabitants for several months. ^48^ Likewise, the infodemic and high prevalence of self-medication are two latent problems in the Peruvian population. ^11, 49^ Both of these behaviors could increase the mortality of COVID-19 by providing false security and by people not attending health services in a timely manner, leading to a worse prognosis. The same occurs with the use of corticosteroids in the early and mild stages of the disease. ^50^ On the other hand, the high rate of job informality and poverty in Peru has also aggravated the situation and could explain the poor adherence to quarantine and the failure to implement community mitigation strategies. ^51^ This was reflected in a high mortality rate, especially in vulnerable groups. ^52^

This study has limitations: 1) The population included in the study were patients insured by social security, which is made up of salaried workers and their families, which may not be representative of the entire country due to its socioeconomic characteristics; 2) There was a high percentage of missing values in certain relevant laboratory markers, which limited their evaluation in the multivariate model. However, relevant and useful markers described in the literature were included; 3) There are laboratory markers of immune response such as cytokines that could not be measured in the present analysis. Despite these limitations, this study represents one of the first reports from Peru, ^12, 53^ a country that became the global epicenter of the pandemic and leaves many lessons to be dealt with in the future to improve the failures of the health system and management of evidence-based disease.

## Conclusions

The risk factors found are consistent with what has been described in the literature and allow the identification of vulnerable groups in whom monitoring and early identification of symptoms should be prioritized. Likewise, the findings of our study describe what happened during the first stage of the pandemic in Peru, highlighting the late arrival to receiving medical attention, as well as the lack of ICU beds, leading to a high incidence of mortality. In addition, the number of ICU beds, hospital beds and access to oxygen in the population should be improved in order to reduce mortality. Finally, evidence-based treatment schemes must be implemented to combat the infodemic and self-medication in the population of Peru.

## Data Availability

Figshare: Database including information of COVID-19 patients from Peru. DOI: https://doi.org/10.6084/m9.figshare.14170955.v1. ^54^ This project contains the following underlying data:
- .xls file containing the information of COVID-19 patients from Peru. Data are available under the terms of the Creative Commons Zero "No rights reserved" data waiver (CC BY 4.0 Public domain dedication).
