# Supplementary material for: Risk factors for mortality in hospitalized patients with COVID-19 from three hospitals in Peru: a retrospective cohort study
Source: F1000Res. 2021 Mar 19;10:224. [Version 1] doi: 10.12688/f1000research.51474.1 (PMC8207806; doi:10.12688/f1000research.51474.1)
Supplement: Peer review history

# Peer review: Reviewer response for version 1

**DOI:** 10.5256/f1000research.54648.r83302

**Published:** 2021-06-15

**Authors:** Miguel Oswaldo Cadena Sanabria

**Affiliations:** 1 Internal Medicine Department, University Hospital of Santander, Industrial University of Santander, Bucaramanga, Colombia

The present study includes clinical and sociodemographic data in a cohort of Peruvian adults with COVID-19 and describes the factors associated with mortality. The authors demonstrated the concordance of the variables and medical conditions associated with higher mortality. It is a population with high mortality, less than 80% of the patients received steroids according to the recovery study protocol; however, given that the population was evaluated between March to May 2020, there was still uncertainty in effective treatments. Remarkably, the male gender was not associated with a higher risk of dying from COVID-19. There is adequate methodology and coherence with the results and conclusions.

 It should be noted that only 3.4% of the cases were confirmed with a positive RT-PCR test. Serological diagnoses (IgM/IgG) tend to be later and may be related to an increased risk of mortality. Today, antigenic tests are available, which also allow a faster diagnosis.

 The Cox regression model is adequate for the purpose of the study. The authors adequately recognize biases and limitations, as well as the number of losses in paraclinical variables.

 This study is based on a hospital population with a census sample (it included all the cases that met the inclusion criteria). This limits the external validity or generalizability of the results. Mortality is oversized since it does not represent the risk of lethality in the general population with COVID-19. Another aspect associated with higher mortality could have been the limitation in admission to the intensive care unit, only 3.3% of the patients were admitted. These key aspects are recognized by the researchers and are part of the paper.

Is the work clearly and accurately presented and does it cite the current literature?

Yes

If applicable, is the statistical analysis and its interpretation appropriate?

Yes

Are all the source data underlying the results available to ensure full reproducibility?

Yes

Is the study design appropriate and is the work technically sound?

Yes

Are the conclusions drawn adequately supported by the results?

Yes

Are sufficient details of methods and analysis provided to allow replication by others?

Yes

Reviewer Expertise:

Internal Medicine, Geriatrics, Medical Education

I confirm that I have read this submission and believe that I have an appropriate level of expertise to confirm that it is of an acceptable scientific standard.

# Peer review: Reviewer response for version 1

**DOI:** 10.5256/f1000research.54648.r84549

**Published:** 2021-05-25

**Authors:** José Fernando Gómez Montes

**Affiliations:** 1 Research Group in Geriatrics and Gerontology, Faculty of Health Sciences, Universidad de Caldas, Manizales, Colombia

**General comment**

 This is a retrospective cohort study about of the risk factors for mortality in patients hospitalized for COVID-19 in three hospitals in Peru in 2020. The topic of the manuscript is appropriate for the Journal. It is of high interest to investigators and clinicians. Their findings provide valuable information about risk factors associated with deaths in developing countries; these results can be considerably useful for epidemiological description of the disease in terms of person-level risk. Minor essential revisions are necessary.

**Minor essential revisions**

**Tittle: **

- The title is accurate and sufficiently descriptive of the content.
- The title is consistent with the presented problem and reflects the main message of the study.

**Abstract:**

- Abstract: Concise and specific.
- Main objective of the review is presented.
- Abstract highlights the contribution of this work.

**Introduction:**

- The background of the study is clear and helpful to readers unfamiliar with the subject.
- The purpose of the article is clearly presented.
- Information is clearly provided.

**Methods:**

- The approach and study design are appropriateness.
- More information about serological and molecular test for Covid-19 is desirable.
- What are the requirements to be hospitalized in ICU? Please provide what ICU admission criteria were used.

**Discussion:**

- The discussion is relevant.
- Conclusions are logically valid and justified by evidence about the main theme proposed.

**References:**

- There were 54 and all are appropriate and relevant. However check references carefully; for example references 16.27.36.47.48.50. 51.53.54 are no complete; please provide them according to journal requirements. 

**Tables and figures:**

- Three tables and three figures are shown. All of them clear and well designed.

Thanks for letting me review this manuscript. This could be a nice paper.

 Level of interest: An article whose findings are important to those with closely related research interests.

 Quality of written English: Well.

 Statistical review: No.

 Declaration of competing interests: I declare that I have no competing interest.

Is the work clearly and accurately presented and does it cite the current literature?

Yes

If applicable, is the statistical analysis and its interpretation appropriate?

Yes

Are all the source data underlying the results available to ensure full reproducibility?

Yes

Is the study design appropriate and is the work technically sound?

Yes

Are the conclusions drawn adequately supported by the results?

Yes

Are sufficient details of methods and analysis provided to allow replication by others?

Yes

Reviewer Expertise:

Geriatrics, Falls, Frailty Geriatric syndromes

I confirm that I have read this submission and believe that I have an appropriate level of expertise to confirm that it is of an acceptable scientific standard.
